# Supplementary material for: Computational Analysis of SAM Analogs as Methyltransferase Inhibitors of nsp16/nsp10 Complex from SARS-CoV-2
Source: Int J Mol Sci. 2022 Nov 12;23(22):13972. doi: 10.3390/ijms232213972 (PMC9697258; doi:10.3390/ijms232213972)
Supplement: Supplementary file 1 [file ijms-23-13972-s001.zip › Supplementary Materials.pdf]

Computational Analysis of SAM Analogs as Methyltransferase Inhibitors of nsp16/nsp10 complex from SARS-CoV-2

Alessandra M. Balieiro, Eduarda L. S. Anunciação, Clauber H. S. Costa, Wesam S. Qayed and José Rogério A. Silva

Supplementary Materials

Figure S1. Conformations acquired by the nsp16 protein during 200 ns of MD simulations for 1a, 2b and 4c systems. PCA plots (left) from initial (blue) to 200 ns (red) structures. Initial, intermediate, and final 3D conformations are highlighted in orange, green and purple, respectively.

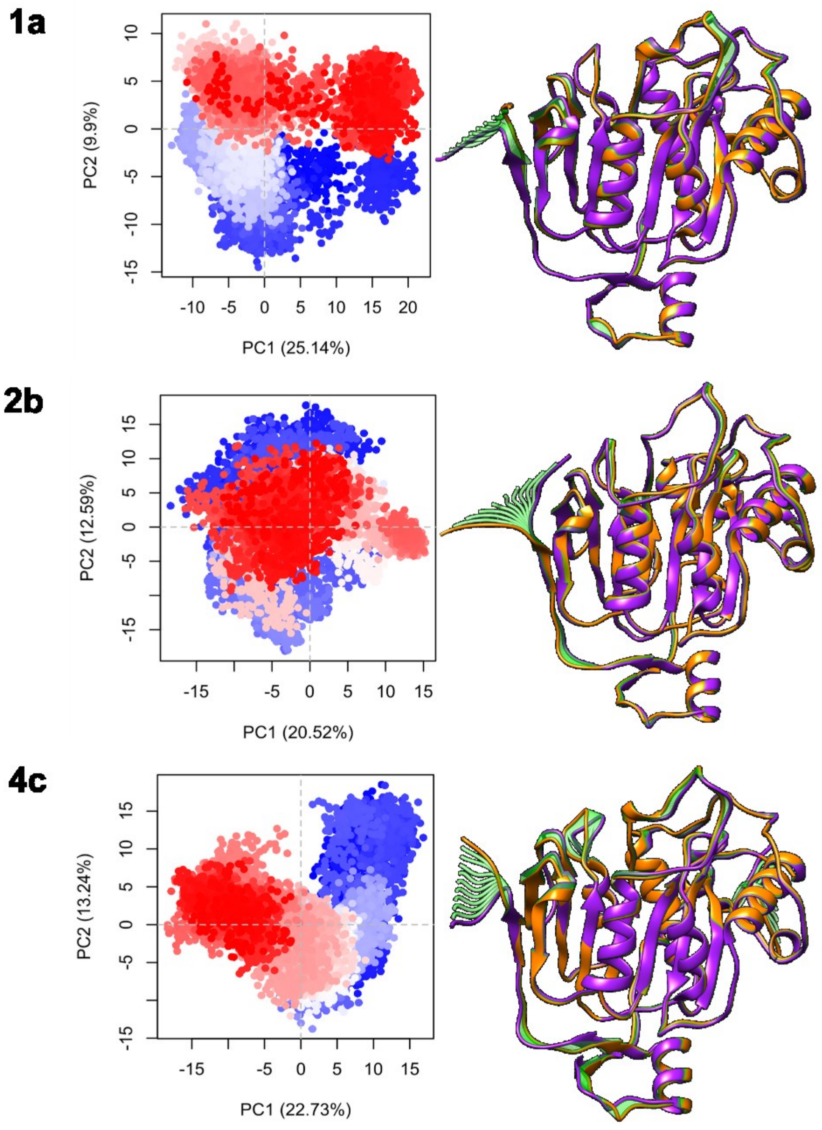

Figure S2. Labeled atoms of 2D structures of simulated compounds.

SAM

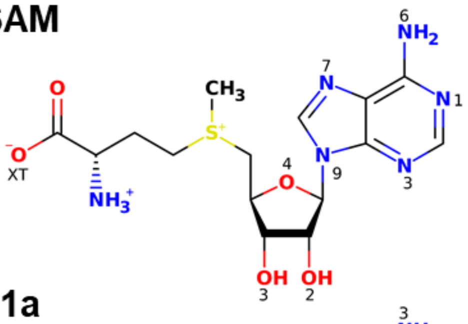

SFG

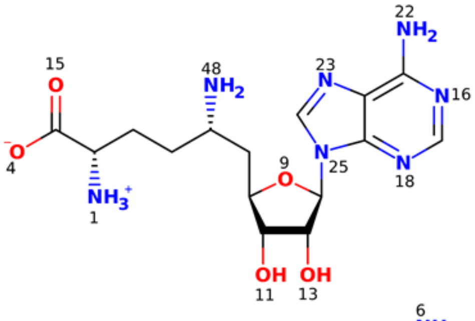

1a

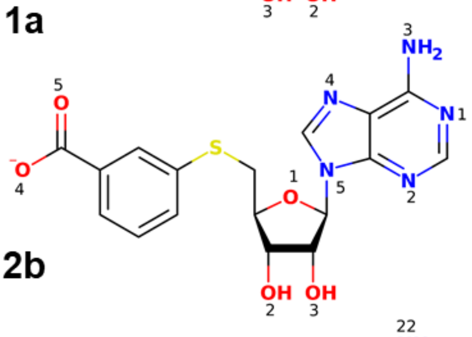

2a

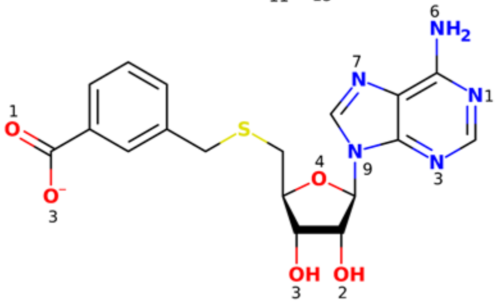

2b

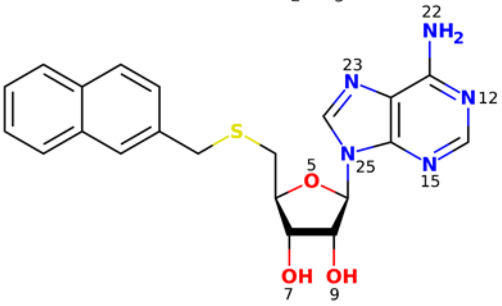

4c

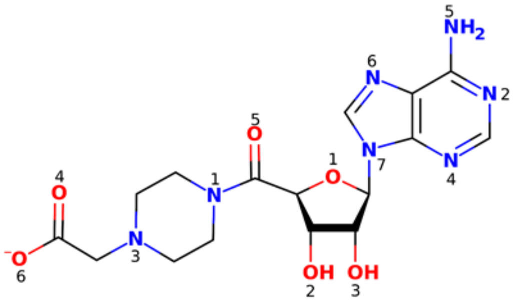

.

Table S1. RMSF values (in Å) for all nsp16 systems.

Residue	Index	1a	2a	2b	4c	SAM	SFG
SER	1	3.05	4.13	4.04	6.06	4.65	4.85
SER	2	2.03	2.19	2.08	3.97	2.46	2.17
GLN	3	1.53	1.22	1.31	3.49	1.56	1.33
ALA	4	1.24	1.03	1.09	1.22	1.13	1.05
TRP	5	0.94	0.89	0.83	0.98	0.89	0.86
GLN	6	0.54	0.55	0.57	0.56	0.54	0.51
PRO	7	0.42	0.44	0.45	0.42	0.41	0.41
GLY	8	0.41	0.44	0.45	0.42	0.41	0.40
VAL	9	0.42	0.42	0.45	0.46	0.40	0.41
ALA	10	0.47	0.48	0.47	0.50	0.45	0.45
MET	11	0.43	0.46	0.44	0.43	0.43	0.42
PRO	12	0.57	0.60	0.59	0.56	0.58	0.60
ASN	13	0.66	0.67	0.68	0.64	0.69	0.70
LEU	14	0.72	0.81	0.74	0.69	0.81	0.79
TYR	15	0.59	0.72	0.58	0.55	0.72	0.74
LYS	16	0.64	0.77	0.61	0.60	0.79	0.82

MET	17	0.73	0.87	0.70	0.69	0.84	0.93
GLN	18	0.65	0.81	0.63	0.60	0.71	0.73
ARG	19	0.65	0.69	0.65	0.62	0.69	0.71
MET	20	0.61	0.63	0.61	0.59	0.64	0.61
LEU	21	0.73	0.71	0.74	0.70	0.76	0.74
LEU	22	0.67	0.65	0.69	0.64	0.69	0.65
GLU	23	0.71	0.68	0.76	0.68	0.81	0.66
LYS	24	0.65	0.65	0.77	0.65	0.79	0.57
CYS	25	0.61	0.57	0.70	0.57	0.65	0.52
ASP	26	0.66	0.63	0.70	0.56	0.60	0.60
LEU	27	0.64	0.63	0.70	0.54	0.62	0.60
GLN	28	1.00	0.86	0.99	0.79	0.94	0.87
ASN	29	1.06	0.72	0.80	0.65	0.85	0.58
TYR	30	0.88	0.61	0.62	0.52	0.71	0.56
GLY	31	1.46	0.93	1.22	0.86	1.42	0.94
ASP	32	1.18	0.74	0.91	0.76	1.03	0.75
SER	33	1.19	0.86	0.93	0.81	1.02	0.87
ALA	34	0.96	0.80	0.86	0.81	0.88	0.82
THR	35	0.70	0.75	0.75	0.77	0.72	0.76
LEU	36	0.58	0.62	0.61	0.62	0.62	0.68
PRO	37	0.72	0.84	0.79	0.81	0.84	1.00
LYS	38	0.88	1.18	0.97	1.02	1.09	1.28
GLY	39	0.80	1.05	0.89	0.93	0.99	1.22
ILE	40	0.51	0.54	0.54	0.64	0.58	0.71
MET	41	0.51	0.51	0.55	0.67	0.59	0.68
MET	42	0.42	0.52	0.47	0.54	0.45	0.53
ASN	43	0.45	0.51	0.46	0.48	0.44	0.57
VAL	44	0.37	0.41	0.38	0.41	0.37	0.45
ALA	45	0.37	0.43	0.39	0.44	0.37	0.40
LYS	46	0.39	0.46	0.41	0.40	0.35	0.41
TYR	47	0.39	0.45	0.35	0.35	0.32	0.35
THR	48	0.34	0.37	0.34	0.33	0.33	0.33
GLN	49	0.34	0.37	0.35	0.32	0.33	0.32
LEU	50	0.37	0.41	0.38	0.35	0.35	0.35
CYS	51	0.38	0.43	0.38	0.36	0.35	0.37
GLN	52	0.39	0.42	0.40	0.37	0.36	0.38
TYR	53	0.41	0.45	0.42	0.41	0.40	0.40
LEU	54	0.42	0.47	0.42	0.41	0.39	0.41
ASN	55	0.45	0.54	0.47	0.45	0.42	0.47
THR	56	0.49	0.58	0.49	0.50	0.46	0.47
LEU	57	0.46	0.53	0.44	0.46	0.43	0.46
THR	58	0.48	0.54	0.48	0.51	0.46	0.49
LEU	59	0.55	0.64	0.60	0.66	0.48	0.54
ALA	60	0.52	0.60	0.49	0.52	0.44	0.54
VAL	61	0.45	0.51	0.45	0.47	0.42	0.47

PRO	62	0.48	0.51	0.49	0.50	0.47	0.56
TYR	63	0.55	0.59	0.65	0.62	0.57	0.65
ASN	64	0.58	0.63	0.65	0.61	0.63	0.67
MET	65	0.45	0.45	0.44	0.42	0.47	0.48
ARG	66	0.41	0.42	0.48	0.38	0.46	0.45
VAL	67	0.36	0.37	0.34	0.34	0.36	0.37
ILE	68	0.39	0.38	0.36	0.37	0.36	0.39
HID	69	0.39	0.35	0.35	0.36	0.35	0.40
PHE	70	0.39	0.38	0.36	0.53	0.33	0.41
GLY	71	0.48	0.48	0.43	0.68	0.35	0.49
ALA	72	0.51	0.44	0.43	0.52	0.36	0.45
GLY	73	0.69	0.64	0.51	0.64	0.43	0.55
SER	74	0.62	0.61	0.78	0.89	0.70	0.82
ASP	75	0.66	0.79	0.97	1.12	0.82	1.11
LYS	76	0.56	0.73	0.94	0.97	0.72	0.97
GLY	77	0.56	0.66	0.77	1.10	0.61	0.77
VAL	78	0.46	0.55	0.55	0.63	0.52	0.55
ALA	79	0.45	0.45	0.47	0.46	0.46	0.52
PRO	80	0.44	0.46	0.45	0.46	0.46	0.49
GLY	81	0.45	0.45	0.43	0.42	0.40	0.45
THR	82	0.41	0.43	0.39	0.36	0.37	0.42
ALA	83	0.42	0.46	0.41	0.40	0.40	0.42
VAL	84	0.36	0.39	0.34	0.36	0.34	0.36
LEU	85	0.38	0.43	0.36	0.35	0.36	0.37
ARG	86	0.42	0.46	0.43	0.40	0.44	0.41
GLN	87	0.42	0.63	0.44	0.42	0.42	0.41
TRP	88	0.37	0.40	0.38	0.37	0.38	0.38
LEU	89	0.40	0.42	0.40	0.41	0.41	0.41
PRO	90	0.53	0.55	0.55	0.58	0.55	0.55
THR	91	0.75	0.89	0.83	0.84	0.81	0.90
GLY	92	0.70	0.97	0.85	0.89	0.81	0.82
THR	93	0.48	0.55	0.50	0.51	0.51	0.51
LEU	94	0.44	0.46	0.42	0.42	0.46	0.46
LEU	95	0.39	0.43	0.39	0.38	0.41	0.41
VAL	96	0.41	0.43	0.38	0.39	0.41	0.41
ASP	97	0.39	0.39	0.35	0.38	0.37	0.42
SER	98	0.42	0.46	0.39	0.44	0.41	0.47
ASP	99	0.54	0.50	0.43	0.50	0.37	0.49
LEU	100	0.73	0.61	0.60	0.66	0.49	0.62
ASN	101	0.95	1.14	0.73	0.98	0.69	0.82
ASP	102	0.89	1.03	0.79	0.94	0.74	0.74
PHE	103	0.53	0.67	0.60	0.59	0.51	0.58
VAL	104	0.59	0.59	0.63	0.69	0.58	0.63
SER	105	0.48	0.50	0.50	0.48	0.46	0.51
ASP	106	0.63	0.53	0.52	0.59	0.48	0.50

ALA	107	0.46	0.48	0.45	0.46	0.45	0.47
ASP	108	0.50	0.52	0.50	0.51	0.52	0.53
SER	109	0.53	0.57	0.53	0.53	0.53	0.56
THR	110	0.46	0.54	0.48	0.47	0.49	0.52
LEU	111	0.68	0.59	0.57	0.61	0.57	0.63
ILE	112	0.78	0.89	0.59	0.70	0.54	0.66
GLY	113	0.88	0.74	0.65	0.77	0.59	0.75
ASP	114	0.66	0.56	0.55	0.69	0.47	0.58
CYS	115	0.64	0.65	0.58	0.73	0.50	0.61
ALA	116	0.68	0.64	0.67	0.83	0.58	0.67
THR	117	0.76	0.73	0.68	0.72	0.60	0.68
VAL	118	0.70	0.70	0.64	0.59	0.57	0.64
HIP	119	0.65	0.71	0.70	0.62	0.61	0.66
THR	120	0.58	0.57	0.54	0.52	0.53	0.57
ALA	121	0.79	0.68	0.69	0.75	0.77	0.70
ASN	122	0.59	0.56	0.51	0.51	0.60	0.53
LYS	123	0.56	0.49	0.45	0.45	0.55	0.47
TRP	124	0.40	0.41	0.39	0.37	0.39	0.43
ASP	125	0.36	0.36	0.36	0.34	0.37	0.39
LEU	126	0.36	0.36	0.35	0.33	0.35	0.36
ILE	127	0.38	0.40	0.39	0.33	0.37	0.42
ILE	128	0.44	0.43	0.40	0.35	0.36	0.43
SER	129	0.53	0.54	0.50	0.36	0.35	0.41
ASP	130	0.52	0.51	0.58	0.39	0.33	0.44
MET	131	0.63	0.48	0.57	0.44	0.36	0.40
TYR	132	0.54	0.45	0.55	0.43	0.42	0.41
ASP	133	0.68	0.58	0.92	0.65	0.79	0.60
PRO	134	0.75	0.77	1.45	0.79	1.26	1.06
LYS	135	0.79	0.91	1.17	0.99	1.32	1.48
THR	136	0.69	0.81	1.54	1.01	1.45	1.22
LYS	137	0.85	1.37	1.84	1.05	1.74	1.81
ASN	138	1.17	1.36	1.89	1.29	2.34	1.75
VAL	139	1.60	2.32	1.98	1.42	2.56	1.95
THR	140	1.73	2.50	1.97	1.52	2.43	2.07
LYS	141	1.56	1.55	1.56	1.16	1.46	1.57
GLU	142	1.19	1.02	1.05	0.93	0.99	1.07
ASN	143	0.69	0.67	0.65	0.62	0.62	0.64
ASP	144	0.60	0.63	0.58	0.59	0.59	0.59
SER	145	0.50	0.53	0.50	0.49	0.48	0.48
LYS	146	0.53	0.53	0.49	0.46	0.46	0.45
GLU	147	0.76	0.84	0.73	0.70	0.65	0.71
GLY	148	0.97	1.16	0.96	1.31	0.86	0.89
PHE	149	0.65	0.64	0.60	0.56	0.49	0.51
PHE	150	0.49	0.52	0.51	0.44	0.44	0.46
THR	151	0.53	0.69	0.63	0.52	0.54	0.56

TYR	152	0.60	0.66	0.59	0.45	0.49	0.54
ILE	153	0.63	0.61	0.53	0.44	0.44	0.48
CYS	154	0.56	0.54	0.48	0.46	0.44	0.46
GLY	155	0.60	0.57	0.54	0.47	0.48	0.52
PHE	156	0.69	0.66	0.59	0.46	0.50	0.54
ILE	157	0.75	0.72	0.61	0.47	0.53	0.56
GLN	158	0.64	0.68	0.57	0.50	0.54	0.56
GLN	159	0.61	0.61	0.56	0.52	0.54	0.56
LYS	160	0.43	0.46	0.44	0.41	0.42	0.45
LEU	161	0.39	0.41	0.39	0.38	0.39	0.43
ALA	162	0.40	0.44	0.40	0.39	0.39	0.44
LEU	163	0.41	0.46	0.41	0.41	0.39	0.45
GLY	164	0.39	0.43	0.40	0.39	0.38	0.43
GLY	165	0.42	0.42	0.42	0.43	0.37	0.43
SER	166	0.34	0.35	0.34	0.34	0.33	0.35
VAL	167	0.34	0.37	0.34	0.34	0.33	0.34
ALA	168	0.36	0.39	0.33	0.32	0.32	0.33
ILE	169	0.35	0.38	0.35	0.32	0.33	0.33
LYN	170	0.35	0.39	0.37	0.32	0.32	0.35
ILE	171	0.36	0.35	0.35	0.32	0.31	0.32
THR	172	0.37	0.34	0.36	0.33	0.34	0.34
GLU	173	0.43	0.41	0.40	0.40	0.40	0.37
HID	174	0.44	0.47	0.42	0.42	0.42	0.38
SER	175	0.48	0.43	0.39	0.37	0.36	0.36
TRP	176	0.44	0.41	0.40	0.37	0.36	0.36
ASN	177	0.44	0.44	0.41	0.38	0.37	0.37
ALA	178	0.47	0.47	0.45	0.43	0.42	0.43
ASP	179	0.51	0.53	0.50	0.46	0.45	0.46
LEU	180	0.50	0.54	0.47	0.43	0.41	0.41
TYR	181	0.43	0.46	0.43	0.41	0.40	0.40
LYS	182	0.48	0.48	0.47	0.45	0.44	0.44
LEU	183	0.53	0.54	0.48	0.44	0.44	0.43
MET	184	0.47	0.49	0.47	0.46	0.47	0.45
GLY	185	0.55	0.60	0.55	0.53	0.55	0.52
HID	186	0.50	0.52	0.47	0.45	0.45	0.44
PHE	187	0.42	0.44	0.40	0.41	0.40	0.41
ALA	188	0.44	0.45	0.42	0.42	0.41	0.43
TRP	189	0.46	0.47	0.45	0.45	0.44	0.44
TRP	190	0.37	0.38	0.36	0.37	0.36	0.36
THR	191	0.35	0.36	0.36	0.36	0.35	0.35
ALA	192	0.35	0.37	0.36	0.34	0.35	0.35
PHE	193	0.35	0.37	0.38	0.35	0.37	0.35
VAL	194	0.37	0.39	0.40	0.38	0.38	0.32
THR	195	0.39	0.46	0.53	0.41	0.38	0.35
ASN	196	0.36	0.42	0.41	0.41	0.36	0.36

VAL	197	0.42	0.44	0.40	0.41	0.38	0.41
ASN	198	0.48	0.52	0.57	0.45	0.42	0.45
ALA	199	0.46	0.51	0.47	0.47	0.48	0.46
SER	200	0.45	0.51	0.49	0.42	0.44	0.44
SER	201	0.42	0.40	0.53	0.51	0.40	0.43
SER	202	0.44	0.41	0.66	0.52	0.58	0.47
GLU	203	0.43	0.47	0.46	0.44	0.39	0.45
ALA	204	0.44	0.44	0.42	0.41	0.41	0.44
PHE	205	0.38	0.39	0.40	0.34	0.37	0.38
LEU	206	0.37	0.38	0.36	0.35	0.34	0.37
ILE	207	0.38	0.41	0.37	0.38	0.38	0.38
GLY	208	0.42	0.48	0.41	0.42	0.42	0.42
CYS	209	0.40	0.43	0.39	0.41	0.39	0.40
ASN	210	0.38	0.41	0.39	0.39	0.35	0.39
TYR	211	0.37	0.40	0.37	0.37	0.35	0.38
LEU	212	0.45	0.46	0.44	0.43	0.42	0.45
GLY	213	0.66	0.67	0.65	0.63	0.62	0.63
LYS	214	0.89	0.94	0.90	0.89	0.90	0.86
PRO	215	0.83	0.90	0.87	0.79	0.83	0.80
ARG	216	0.89	0.98	0.99	0.85	0.94	0.86
GLU	217	0.86	0.88	0.90	0.81	0.96	0.82
GLN	218	0.89	0.95	0.98	0.88	0.98	0.85
ILE	219	0.57	0.61	0.63	0.57	0.67	0.58
ASP	220	0.59	0.63	0.63	0.56	0.65	0.60
GLY	221	0.58	0.63	0.66	0.58	0.66	0.61
TYR	222	0.54	0.57	0.61	0.56	0.61	0.58
VAL	223	0.46	0.47	0.49	0.46	0.53	0.47
MET	224	0.39	0.39	0.41	0.39	0.43	0.39
HIE	225	0.38	0.38	0.40	0.38	0.39	0.38
ALA	226	0.42	0.41	0.44	0.40	0.42	0.41
ASN	227	0.40	0.40	0.42	0.39	0.42	0.40
TYR	228	0.37	0.38	0.41	0.36	0.39	0.38
ILE	229	0.42	0.41	0.43	0.39	0.41	0.39
PHE	230	0.47	0.46	0.48	0.45	0.45	0.44
TRP	231	0.45	0.44	0.48	0.42	0.45	0.42
ARG	232	0.45	0.42	0.46	0.40	0.43	0.41
ASN	233	0.55	0.52	0.56	0.50	0.52	0.50
THR	234	0.61	0.60	0.66	0.57	0.61	0.57
ASN	235	0.69	0.72	0.75	0.62	0.71	0.64
PRO	236	0.86	1.05	0.96	0.78	0.82	0.83
ILE	237	0.75	0.73	0.67	0.62	0.64	0.65
GLN	238	0.84	0.81	0.77	0.71	0.77	0.75
LEU	239	0.51	0.56	0.53	0.54	0.54	0.50
SER	240	0.51	0.52	0.54	0.56	0.52	0.48
SER	241	0.50	0.50	0.51	0.53	0.50	0.48

TYR	242	0.56	0.52	0.52	0.52	0.54	0.52
SER	243	0.48	0.47	0.48	0.47	0.47	0.47
LEU	244	0.47	0.49	0.51	0.52	0.51	0.52
PHE	245	0.85	0.85	0.88	0.89	0.87	0.87
ASP	246	1.17	1.22	1.29	1.20	1.20	1.18
MET	247	0.58	0.64	0.63	0.65	0.61	0.61
SER	248	0.72	0.88	0.77	0.81	0.74	0.78
LYS	249	0.77	1.00	0.82	0.87	0.81	0.82
PHE	250	0.49	0.61	0.51	0.54	0.50	0.50
PRO	251	0.52	0.56	0.52	0.54	0.51	0.55
LEU	252	0.51	0.52	0.50	0.50	0.50	0.53
LYS	253	0.83	0.91	0.88	0.94	0.80	0.91
LEU	254	1.14	1.23	1.00	1.07	1.16	1.19
ARG	255	1.21	1.36	1.18	1.24	1.23	1.32
GLY	256	1.74	1.93	1.55	1.26	1.75	1.89
THR	257	0.64	0.70	0.61	0.56	0.63	0.71
ALA	258	0.58	0.66	0.58	0.56	0.61	0.63
VAL	259	0.56	0.61	0.58	0.58	0.62	0.63
MET	260	0.68	0.77	0.77	0.73	0.75	0.77
SER	261	0.91	0.98	0.86	0.88	0.91	0.88
LEU	262	1.61	1.49	1.08	1.47	1.83	1.06
LYS	263	1.72	1.98	1.76	1.51	1.84	1.46
GLU	264	2.46	2.99	2.26	2.03	2.27	1.82
GLY	265	2.45	2.68	2.23	2.00	2.21	1.94
GLN	266	1.81	2.70	1.64	1.47	2.65	1.55
ILE	267	1.27	1.20	1.11	1.04	1.17	1.10
ASN	268	1.31	1.13	1.15	1.02	1.17	1.21
ASP	269	1.40	1.13	1.18	1.03	1.20	1.23
MET	270	1.25	1.06	1.06	0.94	1.09	1.14
ILE	271	0.79	0.79	0.76	0.69	0.76	0.86
LEU	272	0.76	0.74	0.76	0.67	0.73	0.82
SER	273	0.78	0.76	0.76	0.70	0.73	0.85
LEU	274	0.63	0.64	0.62	0.58	0.60	0.74
LEU	275	0.55	0.54	0.53	0.50	0.55	0.60
SER	276	0.68	0.65	0.64	0.60	0.66	0.68
LYS	277	0.82	0.79	0.72	0.68	0.87	0.83
GLY	278	0.72	0.70	0.62	0.61	0.83	0.75
ARG	279	0.61	0.65	0.56	0.55	0.60	0.66
LEU	280	0.48	0.54	0.47	0.46	0.49	0.55
ILE	281	0.41	0.48	0.42	0.41	0.42	0.53
ILE	282	0.45	0.54	0.52	0.48	0.54	0.60
ARG	283	0.55	0.63	0.64	0.61	0.83	0.65
GLU	284	0.69	0.92	0.92	0.95	1.17	0.71
ASN	285	0.88	1.29	1.56	1.51	1.84	1.06
ASN	286	0.89	1.21	1.51	1.20	1.49	0.97

ARG	287	0.88	1.09	1.43	1.05	1.27	0.93
VAL	288	0.68	0.70	0.74	0.73	0.86	0.65
VAL	289	0.72	0.69	0.67	0.70	0.74	0.71
ILE	290	0.66	0.62	0.61	0.59	0.63	0.63
SER	291	0.90	0.77	0.78	0.64	0.72	0.87
SER	292	0.98	0.91	0.87	0.70	0.79	1.04
ASP	293	1.33	1.50	1.30	1.01	0.97	1.50
VAL	294	1.65	3.10	2.35	1.50	1.47	4.02
LEU	295	3.53	4.87	4.06	3.00	3.35	6.08
VAL	296	5.12	6.74	5.49	4.45	4.82	7.77
ASN	297	6.89	8.66	7.88	6.27	6.77	9.57

Table S2. Per-residue decomposition values (kcal/mol) for nsp16 systems.

Residue	Index	1a	2a	2b	4c	SAM	SFG
SER	1	0.00	0.00	0.00	0.00	0.00	0.00
SER	2	0.00	0.00	0.00	0.00	0.00	0.00
GLN	3	0.00	0.00	0.00	0.00	0.00	0.00
ALA	4	0.00	0.00	0.00	0.00	0.00	0.00
TRP	5	0.00	0.00	0.00	0.00	0.00	0.00
GLN	6	0.00	0.00	0.01	0.01	0.01	0.00
PRO	7	0.00	0.00	0.00	0.00	0.00	0.00
GLY	8	0.00	0.00	0.00	0.00	0.00	0.00
VAL	9	0.00	0.00	0.00	0.00	0.00	0.00
ALA	10	0.00	0.00	0.00	0.00	0.00	0.00
MET	11	0.00	0.00	0.00	0.00	0.00	0.00
PRO	12	0.00	0.00	0.00	0.00	0.00	0.00
ASN	13	0.00	0.00	0.00	0.00	0.00	0.00
LEU	14	0.00	0.00	0.00	0.00	0.00	0.00
TYR	15	0.00	0.00	0.00	0.00	0.00	0.00
LYS	16	0.00	0.00	0.00	0.00	0.01	0.00
MET	17	0.00	0.00	0.00	0.00	0.00	0.00
GLN	18	0.00	0.00	0.00	0.00	0.00	0.00
ARG	19	0.00	0.00	0.00	0.00	0.00	0.00
MET	20	0.00	0.00	0.00	0.00	0.00	0.00
LEU	21	0.00	0.00	0.00	0.00	0.00	0.00
LEU	22	0.00	0.00	0.00	0.00	0.00	0.00
GLU	23	0.01	0.00	0.00	0.01	-0.01	0.00
LYS	24	-0.01	0.00	0.00	-0.01	0.02	0.00
CYS	25	0.00	0.00	0.00	0.00	0.00	0.00
ASP	26	0.01	0.01	0.00	0.01	-0.01	0.00
LEU	27	0.00	0.00	0.00	0.00	0.00	0.00
GLN	28	0.00	0.00	0.00	0.00	0.00	0.00
ASN	29	0.00	0.00	0.00	0.00	0.00	0.00
TYR	30	0.00	0.01	0.01	0.01	0.01	0.01

GLY	31	0.00	0.01	0.00	0.01	0.01	0.00
ASP	32	0.02	0.02	0.00	0.02	-0.01	0.01
SER	33	0.00	0.01	0.01	0.01	0.01	0.01
ALA	34	0.00	0.00	0.00	0.00	0.00	0.00
THR	35	0.00	0.00	0.01	0.00	0.01	0.00
LEU	36	0.00	0.00	0.00	-0.01	0.01	0.00
PRO	37	0.00	0.00	0.00	0.00	0.00	0.00
LYS	38	-0.01	-0.01	0.01	-0.01	0.01	0.00
GLY	39	0.01	0.01	0.01	0.01	0.01	0.01
ILE	40	0.02	0.03	0.02	0.01	0.03	0.02
MET	41	-0.10	-0.06	-0.11	-0.25	-0.01	-0.02
MET	42	-0.21	-0.18	-0.22	-0.74	0.01	-0.03
ASN	43	-3.42	-3.49	-0.88	-1.06	-0.73	-0.32
VAL	44	0.05	0.05	0.00	0.05	0.04	-0.01
ALA	45	0.00	0.01	0.01	-0.04	0.06	0.01
LYS	46	-0.49	-0.40	-0.65	-0.57	1.12	-0.14
TYR	47	0.01	-0.01	-0.16	-0.06	-2.23	-0.05
THR	48	0.02	0.02	0.04	0.01	0.04	0.02
GLN	49	0.01	0.01	0.01	0.00	0.02	0.01
LEU	50	0.00	0.00	0.00	-0.02	0.04	0.00
CYS	51	0.01	0.00	0.01	0.01	-0.01	0.00
GLN	52	0.00	0.00	0.00	0.01	0.00	0.00
TYR	53	0.00	0.00	0.00	0.00	0.01	0.00
LEU	54	0.00	0.00	0.00	0.00	0.01	0.00
ASN	55	0.00	0.00	0.00	0.00	0.00	0.00
THR	56	0.00	0.00	0.00	0.00	0.00	0.00
LEU	57	0.00	0.00	0.00	0.00	0.00	0.00
THR	58	0.00	0.00	0.00	0.00	0.00	0.00
LEU	59	0.00	0.00	0.00	0.00	0.00	0.00
ALA	60	0.00	0.00	0.00	0.00	0.00	0.00
VAL	61	0.00	0.00	0.00	0.00	0.00	0.00
PRO	62	0.00	0.00	0.00	0.00	0.00	0.00
TYR	63	0.00	0.00	0.00	0.00	0.00	0.00
ASN	64	0.00	0.00	0.00	0.00	0.00	0.00
MET	65	0.00	0.00	0.00	0.00	0.01	0.00
ARG	66	0.00	0.00	0.01	0.00	0.02	0.01
VAL	67	0.01	0.01	0.01	0.00	0.05	0.01
ILE	68	0.00	0.01	0.01	-0.02	0.07	0.01
HID	69	0.02	-0.01	-0.04	0.03	-1.18	-0.10
PHE	70	-0.25	-0.25	-0.27	-0.23	-0.13	-0.26
GLY	71	-0.33	-0.47	-0.60	-0.27	-3.41	-1.48
ALA	72	-0.22	-0.31	-0.47	-0.56	-0.42	-0.19
GLY	73	0.02	0.62	0.04	-0.80	-1.84	-1.17
SER	74	-6.23	-4.84	-1.04	-5.13	-1.03	-2.05
ASP	75	0.61	0.68	0.48	2.88	-0.13	1.03

LYS	76	-4.26	-10.43	0.06	-6.25	0.09	-0.85
GLY	77	0.00	-0.01	0.02	-0.04	0.08	0.06
VAL	78	-0.02	-0.02	-0.07	0.00	-0.09	-0.13
ALA	79	-0.06	-0.03	-0.05	-0.14	-0.68	0.08
PRO	80	-0.02	-0.01	-0.30	-0.18	-1.21	-0.07
GLY	81	0.01	0.01	-0.01	-0.07	-0.81	0.04
THR	82	0.01	0.01	0.00	0.00	-0.13	-0.02
ALA	83	0.00	0.00	0.01	0.01	0.00	0.00
VAL	84	0.01	0.01	0.01	-0.03	0.08	0.02
LEU	85	0.00	0.00	0.00	-0.01	0.04	0.01
ARG	86	-0.01	-0.01	0.01	0.01	0.02	0.01
GLN	87	0.01	0.01	0.01	0.01	0.01	0.01
TRP	88	-0.01	-0.01	-0.01	0.00	-0.01	0.00
LEU	89	0.00	0.00	0.01	0.00	0.01	0.00
PRO	90	0.00	0.00	0.00	0.00	-0.01	0.00
THR	91	0.00	0.00	0.00	0.00	0.00	0.00
GLY	92	0.00	0.00	0.00	0.00	0.00	0.00
THR	93	0.00	0.00	0.01	0.01	0.01	0.01
LEU	94	0.00	0.00	0.00	0.00	0.01	0.00
LEU	95	0.00	0.01	0.01	-0.01	0.04	0.01
VAL	96	0.00	0.01	0.01	-0.01	0.05	0.01
ASP	97	0.00	0.01	-0.05	-0.03	-0.21	-0.09
SER	98	-0.09	-0.18	-0.19	-0.11	-0.32	-0.23
ASP	99	-5.23	-2.62	-4.01	-8.16	-12.69	-6.14
LEU	100	-2.32	-2.30	-2.58	-2.62	-2.60	-2.86
ASN	101	-0.47	-0.07	-0.06	-0.22	-0.12	-0.08
ASP	102	0.09	0.08	0.03	0.08	-0.01	0.04
PHE	103	-0.04	-0.04	-0.07	-0.05	-0.31	-0.13
VAL	104	0.00	0.00	-0.01	0.00	-0.01	-0.01
SER	105	0.00	0.00	0.00	0.01	0.03	0.01
ASP	106	0.04	0.04	0.01	0.04	-0.01	0.01
ALA	107	0.00	0.00	0.00	0.01	0.02	0.01
ASP	108	0.01	0.01	0.00	0.01	-0.02	0.00
SER	109	0.01	0.01	0.01	0.01	0.01	0.01
THR	110	0.02	0.03	0.04	0.02	0.10	0.05
LEU	111	0.00	-0.01	-0.02	-0.01	-0.02	-0.01
ILE	112	-0.06	-0.04	-0.04	-0.06	-0.02	-0.02
GLY	113	-0.11	-0.08	-0.10	-0.26	-0.10	-0.03
ASP	114	-1.32	-1.19	-1.01	-0.94	-1.80	-1.60
CYS	115	-2.66	-2.54	-2.40	-1.65	-2.48	-2.89
ALA	116	-0.08	-0.05	-0.06	-0.14	-0.07	-0.07
THR	117	-0.03	-0.01	-0.03	-0.05	0.01	-0.01
VAL	118	-0.01	-0.01	-0.02	-0.03	-0.01	-0.02
HIP	119	0.01	0.01	0.02	0.01	0.03	0.02
THR	120	0.00	0.00	0.00	0.00	0.00	0.00

ALA	121	0.00	0.00	0.00	0.00	0.00	0.00
ASN	122	0.00	0.00	0.00	0.00	0.00	0.00
LYS	123	0.00	0.00	0.01	0.00	0.02	0.01
TRP	124	0.00	-0.01	-0.01	0.00	-0.01	-0.01
ASP	125	0.00	0.00	0.00	0.00	-0.02	-0.01
LEU	126	0.01	0.01	0.01	0.00	0.03	0.01
ILE	127	0.00	0.01	0.00	-0.02	0.07	0.00
ILE	128	-0.04	-0.02	-0.05	-0.07	0.10	-0.03
SER	129	-0.17	-0.07	-0.15	-0.26	0.50	-0.06
ASP	130	2.69	0.74	1.21	2.09	-4.42	0.33
MET	131	-3.03	-2.89	-4.09	-4.73	-2.42	-3.45
TYR	132	-1.30	-2.23	-1.22	-1.68	-2.78	-1.60
ASP	133	-0.22	-1.17	-0.31	-0.48	-1.21	-0.30
PRO	134	-0.23	-0.65	-0.21	-0.32	-0.81	-0.41
LYS	135	0.05	0.17	0.08	0.05	0.28	0.10
THR	136	0.00	0.07	0.02	0.03	0.11	0.01
LYS	137	-0.10	0.03	0.03	-0.02	0.19	0.07
ASN	138	0.02	0.02	0.01	0.00	0.01	0.01
VAL	139	0.00	0.00	0.00	0.00	0.00	0.00
THR	140	0.00	0.00	0.00	0.00	0.00	0.00
LYS	141	0.00	0.01	0.01	0.00	0.02	0.02
GLU	142	0.01	0.01	0.00	0.01	-0.01	0.00
ASN	143	0.00	0.00	0.00	0.00	0.00	0.01
ASP	144	0.02	0.01	0.01	0.02	-0.01	0.00
SER	145	0.01	0.01	0.01	0.01	0.01	0.01
LYS	146	0.01	0.02	0.03	0.01	0.06	0.04
GLU	147	0.04	0.04	0.03	0.02	0.01	0.04
GLY	148	0.00	0.02	0.02	0.06	0.06	0.03
PHE	149	-1.51	-1.54	-0.94	-0.41	-1.28	-1.33
PHE	150	-0.05	-0.09	-0.06	-0.04	-0.13	-0.06
THR	151	0.01	0.01	0.00	-0.01	0.00	0.01
TYR	152	-0.06	-0.03	-0.03	0.00	-0.02	-0.04
ILE	153	-0.07	-0.06	-0.03	-0.02	-0.02	-0.03
CYS	154	0.00	-0.01	0.00	0.00	-0.01	0.00
GLY	155	0.00	0.00	0.00	0.00	0.00	0.00
PHE	156	0.00	0.00	0.00	0.00	0.00	0.00
ILE	157	0.00	0.00	0.00	0.00	0.00	0.00
GLN	158	0.01	0.01	0.01	0.00	0.01	0.01
GLN	159	0.01	0.01	0.01	0.00	0.01	0.01
LYS	160	0.00	0.00	0.01	0.00	0.02	0.01
LEU	161	0.00	0.00	0.00	0.00	0.01	0.00
ALA	162	0.00	0.00	0.00	0.00	0.00	0.00
LEU	163	0.00	0.00	0.00	0.00	0.00	0.00
GLY	164	0.00	0.00	0.00	0.00	0.00	0.00
GLY	165	0.00	0.00	0.00	0.00	0.01	0.00

SER	166	0.00	0.00	0.01	0.00	0.01	0.00
VAL	167	0.01	0.01	0.01	0.00	0.05	0.01
ALA	168	-0.02	0.00	-0.01	-0.02	0.02	-0.01
ILE	169	-0.05	-0.01	-0.05	-0.06	0.08	-0.05
LYN	170	-0.05	0.01	-0.40	-0.26	-0.59	-0.28
ILE	171	-0.01	-0.01	-0.01	-0.01	-0.02	-0.02
THR	172	-0.02	0.00	0.00	-0.01	0.02	0.00
GLU	173	0.02	0.03	0.02	0.03	0.00	0.01
HID	174	-0.01	0.01	0.00	0.00	0.05	0.01
SER	175	0.00	0.02	0.02	0.01	0.06	0.01
TRP	176	0.00	-0.01	-0.01	0.00	-0.03	0.00
ASN	177	0.00	0.00	0.00	0.00	0.01	0.01
ALA	178	0.00	0.00	0.00	0.00	0.00	0.00
ASP	179	0.02	0.01	0.01	0.01	0.00	0.01
LEU	180	0.00	0.00	0.00	0.00	0.01	0.00
TYR	181	0.00	0.00	0.00	0.00	-0.01	0.00
LYS	182	0.00	0.00	0.00	0.00	0.01	0.00
LEU	183	0.00	0.00	0.00	0.00	0.01	0.00
MET	184	0.00	0.00	0.00	0.00	0.00	0.00
GLY	185	0.00	0.00	0.00	0.00	0.00	0.00
HID	186	0.00	0.00	0.00	0.00	0.00	0.00
PHE	187	-0.01	-0.01	-0.01	-0.01	-0.01	-0.01
ALA	188	0.00	0.00	0.00	0.00	0.00	0.00
TRP	189	0.00	0.00	0.00	0.00	0.00	0.00
TRP	190	0.00	0.00	0.00	0.00	0.00	0.00
THR	191	0.00	0.00	0.00	0.00	0.00	0.00
ALA	192	0.00	0.01	0.01	0.00	0.02	0.01
PHE	193	-0.01	-0.01	-0.02	-0.01	-0.01	-0.01
VAL	194	0.00	0.01	0.01	-0.01	0.02	0.01
THR	195	0.00	0.01	0.00	-0.03	0.02	0.00
ASN	196	0.01	0.01	0.01	0.00	0.02	0.01
VAL	197	0.01	0.01	0.02	-0.01	0.03	0.01
ASN	198	-0.05	-0.02	-0.02	-0.07	0.02	0.01
ALA	199	0.00	0.00	0.01	0.01	0.00	0.01
SER	200	0.00	0.01	0.01	0.00	0.01	0.01
SER	201	-0.01	0.01	0.00	-0.01	0.02	0.00
SER	202	-0.01	0.01	0.00	-0.02	0.03	-0.01
GLU	203	0.06	0.13	-0.01	0.05	0.01	0.12
ALA	204	0.00	0.00	-0.01	-0.02	0.00	-0.01
PHE	205	0.01	-0.01	0.00	0.04	-0.09	0.00
LEU	206	0.00	0.00	0.00	0.00	0.00	0.00
ILE	207	0.00	0.00	0.00	0.00	0.00	0.00
GLY	208	0.00	0.00	0.00	0.00	0.00	0.00
CYS	209	0.00	0.00	0.00	0.00	0.00	0.00
ASN	210	0.00	0.00	0.00	0.00	0.00	0.00

TYR	211	0.00	0.00	0.00	0.00	0.00	0.00
LEU	212	0.00	0.00	0.00	0.00	0.00	0.00
GLY	213	0.00	0.00	0.00	0.00	0.00	0.00
LYS	214	0.00	0.00	0.00	0.00	0.00	0.00
PRO	215	0.00	0.00	0.00	0.00	0.00	0.00
ARG	216	0.00	0.00	0.00	0.00	0.00	0.00
GLU	217	0.00	0.00	0.00	0.00	-0.01	0.00
GLN	218	0.00	0.00	0.00	0.00	0.00	0.00
ILE	219	0.00	0.00	0.00	0.00	0.00	0.00
ASP	220	0.00	0.00	0.00	0.00	-0.01	0.00
GLY	221	0.00	0.00	0.00	0.00	0.00	0.00
TYR	222	0.00	0.00	0.00	0.00	0.00	0.00
VAL	223	0.00	0.00	0.00	0.00	0.00	0.00
MET	224	0.00	0.00	0.00	0.00	0.00	0.00
HIE	225	0.00	0.00	0.00	0.00	-0.01	0.00
ALA	226	0.00	0.00	0.00	0.00	0.00	0.00
ASN	227	0.00	0.00	0.00	0.00	0.00	0.00
TYR	228	0.00	0.00	0.00	-0.01	0.00	0.00
ILE	229	0.00	0.00	0.00	0.00	0.00	0.00
PHE	230	0.00	0.00	0.00	0.00	0.00	0.00
TRP	231	0.00	0.00	0.00	0.00	0.00	0.00
ARG	232	0.00	0.01	0.03	0.00	0.06	0.03
ASN	233	0.00	0.00	0.00	0.00	0.00	0.00
THR	234	0.00	0.00	0.00	0.00	0.00	0.00
ASN	235	0.00	0.00	0.00	0.00	0.00	0.00
PRO	236	0.00	0.00	0.00	0.00	0.00	0.00
ILE	237	0.00	0.00	0.00	0.00	0.00	0.00
GLN	238	0.00	0.00	0.00	0.00	0.00	0.00
LEU	239	0.00	0.00	0.01	0.00	0.01	0.00
SER	240	0.00	0.00	0.00	0.00	0.00	0.00
SER	241	0.00	0.00	0.00	0.00	0.00	0.00
TYR	242	0.00	0.00	0.00	0.00	0.00	0.00
SER	243	0.00	0.00	0.00	0.00	0.00	0.00
LEU	244	0.00	0.00	0.00	0.00	0.00	0.00
PHE	245	0.00	0.00	0.00	0.00	0.00	0.00
ASP	246	0.01	0.01	0.00	0.01	0.00	0.00
MET	247	0.00	0.00	0.00	0.00	0.00	0.00
SER	248	0.00	0.00	0.00	0.00	0.00	0.00
LYS	249	0.00	0.00	0.00	0.00	0.01	0.00
PHE	250	0.00	0.00	0.00	0.00	0.00	0.00
PRO	251	0.00	0.00	0.00	0.00	0.00	0.00
LEU	252	0.00	0.00	0.00	0.00	0.00	0.00
LYS	253	0.00	0.00	0.00	0.00	0.00	0.00
LEU	254	0.00	0.00	0.00	0.00	0.00	0.00
ARG	255	0.00	0.00	0.00	0.00	0.01	0.00

GLY	256	0.00	0.00	0.00	0.00	0.00	0.00
THR	257	0.00	0.00	0.00	0.00	0.00	0.00
ALA	258	0.00	0.00	0.00	0.00	0.00	0.00
VAL	259	0.00	0.00	0.00	0.00	0.00	0.00
MET	260	0.00	0.00	0.00	0.00	0.00	0.00
SER	261	0.00	0.00	0.00	0.00	0.00	0.00
LEU	262	0.00	0.00	0.00	0.00	0.00	0.00
LYS	263	0.00	0.00	0.00	0.00	0.00	0.00
GLU	264	0.00	0.00	0.00	0.00	0.00	0.00
GLY	265	0.00	0.00	0.00	0.00	0.00	0.00
GLN	266	0.00	0.00	0.00	0.00	0.00	0.00
ILE	267	0.00	0.00	0.00	0.00	0.00	0.00
ASN	268	0.00	0.00	0.00	0.00	0.00	0.00
ASP	269	0.00	0.00	0.00	0.00	0.00	0.00
MET	270	0.00	0.00	0.00	0.00	0.00	0.00
ILE	271	0.00	0.00	0.00	0.00	0.00	0.00
LEU	272	0.00	0.00	0.00	0.00	0.00	0.00
SER	273	0.00	0.00	0.00	0.00	0.00	0.00
LEU	274	0.00	0.00	0.00	0.00	0.00	0.00
LEU	275	0.00	0.00	0.00	0.00	0.00	0.00
SER	276	0.00	0.00	0.00	0.00	0.00	0.00
LYS	277	0.00	0.00	0.00	0.00	0.00	0.00
GLY	278	0.00	0.00	0.00	0.00	0.00	0.00
ARG	279	0.00	0.00	0.00	0.00	0.00	0.00
LEU	280	0.00	0.00	0.00	0.00	0.00	0.00
ILE	281	0.00	0.00	0.00	0.00	0.00	0.00
ILE	282	0.00	0.00	0.00	0.00	0.00	0.00
ARG	283	0.00	0.00	0.00	0.00	0.01	0.01
GLU	284	0.00	0.00	0.00	0.00	0.00	0.00
ASN	285	0.00	0.00	0.00	0.00	0.00	0.00
ASN	286	0.00	0.00	0.00	0.00	0.00	0.00
ARG	287	0.00	0.00	0.00	0.00	0.01	0.00
VAL	288	0.00	0.00	0.00	0.00	0.00	0.00
VAL	289	0.00	0.00	0.00	0.00	0.00	0.00
ILE	290	0.00	0.01	0.01	0.00	0.01	0.01
SER	291	0.00	0.00	0.01	0.00	0.00	0.00
SER	292	0.01	0.01	0.02	0.01	0.01	0.01
ASP	293	0.02	0.01	0.01	0.02	-0.01	0.01
VAL	294	0.00	0.00	0.00	0.00	0.01	0.01
LEU	295	0.00	0.00	0.00	0.00	0.00	0.00
VAL	296	0.00	0.00	0.00	0.00	0.00	0.00
ASN	297	0.01	0.00	0.01	0.01	0.00	0.00
